# Segregation of mtDNA Throughout Human Embryofetal Development: m.3243A > G as a Model System

**DOI:** 10.1002/humu.21417

**Published:** 2010-11-30

**Authors:** Sophie Monnot, Nadine Gigarel, David C Samuels, Philippe Burlet, Laetitia Hesters, Nelly Frydman, René Frydman, Violaine Kerbrat, Benoit Funalot, Jelena Martinovic, Alexandra Benachi, Josué Feingold, Arnold Munnich, Jean-Paul Bonnefont, Julie Steffann

**Affiliations:** 1 Université Paris-Descartes, Unité INSERM U781, and Hopital Necker-Enfants Malades (Assistance Publique-Hopitaux de Paris)Paris, France; 2 Center for Human Genetics Research, Department of Molecular Physiology and Biophysics, Vanderbilt University Medical CenterNashville, Tennessee; 3 Service de gynécologie et médecine de la reproduction, Hôpital Antoine Béclère (Assistance Publique-Hopitaux de Paris)Clamart, France; 4 Service d'histo-embryologie cytogénétique, Hopital Necker-Enfants Malades (Assistance Publique-Hopitaux de Paris)Paris, France; 5 Maternité, Hôpital Necker-Enfants Malades (Assistance Publique-Hopitaux de Paris)Paris, France

**Keywords:** mitochondria, mitochondrial DNA, MELAS, NARP, respiratory chain deficiency, embryo, preimplantation genetic diagnosis

## Abstract

Mitochondrial DNA (mtDNA) mutations cause a wide range of serious diseases with high transmission risk and maternal inheritance. Tissue heterogeneity of the heteroplasmy rate (“mutant load”) accounts for the wide phenotypic spectrum observed in carriers. Owing to the absence of therapy, couples at risk to transmit such disorders commonly ask for prenatal (PND) or preimplantation diagnosis (PGD). The lack of data regarding heteroplasmy distribution throughout intrauterine development, however, hampers the implementation of such procedures. We tracked the segregation of the m.3243A > G mutation (*MT-TL1* gene) responsible for the MELAS syndrome in the developing embryo/fetus, using tissues and cells from eight carrier females, their 38 embryos and 12 fetuses. Mutant mtDNA segregation was found to be governed by random genetic drift, during oogenesis and somatic tissue development. The size of the bottleneck operating for m.3243A > G during oogenesis was shown to be individual-dependent. Comparison with data we achieved for the m.8993T > G mutation (*MT-ATP6* gene), responsible for the NARP/Leigh syndrome, indicates that these mutations differentially influence mtDNA segregation during oogenesis, while their impact is similar in developing somatic tissues. These data have major consequences for PND and PGD procedures in mtDNA inherited disorders. Hum Mutat 32:116–125, 2011. © 2010 Wiley-Liss, Inc.

## Introduction

Mitochondrial DNA (mtDNA, GenBank NC_001807.4) disorders are common diseases with maternal inheritance. Their high clinical variability is explained, at least in part, by variation of the mutant load (heteroplasmy) among individuals, and also across organs and tissues within an individual, so that mutant loads will surpass the tissue specific threshold, leading to the manifestation of symptom [Chinnery et al., 1997; Jeppesen et al., 2006]. The usual severity of these diseases, the recurrence risk, usually high though being difficult to predict at the individual level, in offspring from heteroplasmic carriers [Chinnery et al., 1998], and the absence of efficient therapy, commonly result in a request from at-risk couples for prenatal (PND) or preimplantation diagnosis (PGD). Such procedures are, however, hampered by the lack of data regarding mtDNA segregation during embryofetal development.

In this respect, most available data come from animal studies. Dramatic shifts of mtDNA populations (variant/wild-type mtDNA ratio) between the mother and her offspring in cattle have suggested that a tight bottleneck might account for such a rapid segregation [Laipis et al., 1988]. One important function of this bottleneck could be to ensure that new mutations will either be lost or fixed rapidly within individuals, and thus exposed to selection at the population level [Bergstrom and Pritchard, 1998; Roze et al., 2005]. Studies in mice have identified the bottleneck during oogenesis [Jenuth et al., 1996], through either a reduction of mtDNA content in primordial germ cells (PGC) [Cree et al., 2008], or selection of a small effective number of segregation units for mtDNA [Cao et al., 2007, 2009; Wai et al., 2008]. However, there are no available data so far regarding mutant mtDNA segregation in somatic tissues during embryofetal development in animal models.

In humans, a small number of studies of primary oocytes carrying mtDNA deletions [Marchington et al., 1998] or point mutations [Blok et al., 1997; Brown et al., 2001; Marchington et al., 2010] have suggested that a bottleneck operates during oogenesis. Random genetic drift appeared as the principal mechanism determining the level of mutant mtDNA within primary oocytes from a carrier of the most common mtDNA mutation, m.3243A > G [Brown et al., 2001]. This mutation affects the *MT-TL1* gene (mitochondrially encoded tRNA leucine 1 [UUA/G]; MIM♯ 590050), and is responsible for the MELAS (mitochondrial myopathy, encephalopathy, lactic acidosis, and stroke-like episodes; MIM♯ 540000) [Goto et al., 1992] and diabetes-deafness syndromes (MIM♯ 520000) [Manouvrier et al., 1995; van den Ouweland et al., 1992].

It is so far not known whether m.3243A > G segregation is governed by random drift in somatic tissues during human embryofetogenesis. The few available studies on m.3243A > G carrier fetuses reported a uniform distribution of mutation load among different tissues from two heteroplasmic 24-and 25-week-old human fetuses [Cardaioli et al., 2000; Matthews et al., 1994]. Our group found similar mutant loads in chorionic villi and amniocytes from three heteroplasmic fetuses [Bouchet et al., 2006]. All these data suggest that MELAS mutant molecules do not segregate much between 10 to 25 weeks of gestation.

However, we have so far lacked data on the transmission of m.3243A > G by a carrier to early embryos, and subsequently no information on segregation of this mutation throughout the entire period of human embryofetal development has existed. Thanks to our PGD and PND programs, we collected various tissues and cells at various stages of development in embryos and fetuses from m.3243A > G carriers, and investigated local and temporal variation of the mutant load, using a quantification test designed to assess m.3243A > G mutant load at the single-cell level.

Aggregation of our data in early embryos and fetuses, with those from a primary oocyte study in a m.3243A > G carrier [Brown et al., 2001] indicates that mtDNA segregation is governed by random genetic drift, in germ and somatic cell lines, throughout the entire period of human intrauterine development. The size of the bottleneck operating during oogenesis for m.3243A > G is additionally suggested to vary between individuals. Finally, comparison of the current data with those we previously reported for another common mtDNA mutation, m.8993T > G (*MT-ATP6*, ATP synthase subunit 6 gene; MIM♯ 516060) responsible for the NARP syndrome (Neuropathy, Ataxia, Retinitis Pigmentosa; MIM♯ 551500) [Steffann et al., 2006, 2007] indicates that these two mutations differentially influence mtDNA segregation during oogenesis, while their putative impact on mtDNA segregation appears quite similar in somatic cells during embryofetal development.

## Patients, Materials and Methods

### Patients

This study was approved by the National Ethics Comittee from l'Agence de Biomedecine, and all couples gave informed consent for DNA analyses in themselves and their embryos/fetuses.

PND and/or PGD analyses were carried out in eight individuals from seven unrelated families, heteroplasmic for the MELAS m.3243A > G mutation. The questionable predictive value of a fetal mutant load for the postnatal outcome was emphasized to all of the couples. In such situations, counseling necessarily refered to postnatal data, attempting to correlate clinical outcome and mutant load [Chinnery et al., 1997; Uusimaa et al., 2007; Whittaker et al., 2009], and to our personal experience with PGD/PND for another mtDNA disorder, that is, NARP [Gigarel et al., 2005; Steffann et al., 2006, 2007] and with PND for MELAS [Bouchet et al., 2006]. Accordingly, we postulated that mutant loads over 60% during prenatal life might be predictive of a serious disorder, whereas a mutant load below 30% might hopefully be associated with absence or low severity of the disease. The most complex situation was a fetal heteroplasmy value in the intermediate range (30–60%), a common finding for the MELAS m.3243A > G mutation in the postnatal period. There is indeed no available data correlating intermediate mutant load in the prenatal period with the postnatal outcome.

Seven of the eight couples (couples 2 to 8) elected to resort to a conventional PND approach at first. Couple 1 declined the option of pregnancy termination (TOP), and thus directly went to PGD, that was also offered to couple 2, who had previously experienced 2 TOP after PND. Both couples were informed that, at this early embryonic stage (day 3), a number of issues remained unsolved, precluding any conclusive prediction on the pre/postnatal outcome of a carrier embryo. These couples were offered a PND at 14 gestation weeks (GW) to confirm the PGD result in case of pregnancy.

### Materials

#### Postnatal period

Mutant loads from carrier females were assessed from blood, and when available, urine and oral mucosa samples. Single lymphocytes were isolated from patients 1, 2, 3, 5, and 14 as already described [Gigarel et al., 2005].

#### Early embryos

Patients 1 and 2 were subjected to one and three separate PGD cycles, respectively. A standard in vitro fertilization protocol was performed and oocytes were collected and fertilized by intracytoplasmic sperm injection (ICSI).

A total of 38 embryos were analyzed. Twenty-three of these embryos were of appropriate quality, and were therefore submitted to a two-blastomere biopsy at post-ICSI day 3 for mutant load assessment, as previously described [Gigarel et al., 2004]. Briefly, under control by a binocular microscope, the blastomeres were rinsed twice in a drop of PBS supplemented with 0.1% polyvinyl alcohol (Sigma-Aldrich, France), using a mouth-controlled, finely pulled glass pipette before being transferred into a transparent microcentrifuge tube containing 3 µl of lysis buffer [Cui et al., 1989]. A small volume of biopsy medium was used as a PCR negative control for each embryo in order to detect any contamination by exogenous DNA. In the 15 remaining embryos, assessment of the mutant load was performed on the embryo in toto. All blastomeres were separately analyzed, when possible (couple 1: embryos 1–3, and couple 2: embryo 13). Embryo 10 from couple 2 was cultured up to the blastocyst stage (day 5), enabling a trophectoderm biopsy as described elsewhere [McArthur et al., 2005]. The trophectoderm and inner cell mass were analyzed separately.

#### Late embryos and fetuses

Prenatal diagnosis was performed in 12 fetuses from 7 carrier women. Fetus 2c resulted from embryo 7 and 11 transfer after PGD (patient 2), whereas the remaining ones were conceived naturally. Chorionic villi (CV, *n* = 8), amniotic fluids 1 (AF1, *n* = 6), and 2 (AF2, *n* = 1), were sampled at 10, 14, and 30 weeks of gestation (GW), respectively. Individual fetal cells were isolated from CV and AF samples from six fetuses, as already described [Steffann et al., 2007], and analyzed separately, to investigate the intercellular variation of heteroplasmy. Cord blood was collected from three fetuses at birth (fetuses 2c, 3, and 4). Postmortem analyses of various tissues were carried out in two 12-week-old fetuses (fetuses 2a and 2b) and one 19-week-old fetus (5b) after TOP, and in a 20-week-old miscarriage product (fetus 7a). Parental informed consent was obtained for these postmortem analyses. Fetal tissues were carefully dissected, and small tissue biopsies were analyzed separately. Multiple samples were taken from two term placentas (fetuses 2c and 3), and two 12GW placentas (fetuses 2a and b).

### Methods

#### DNA extraction

DNA was extracted from blood and tissues, using the Nucleon Bacc3 kit (Amersham Biosciences, UK), and a classical phenol extraction method, respectively.

#### Quantitative analysis of the m.3243A > G mutant load

MELAS mutant load was quantified using a semiquantitative fluorescent PCR-*HaeIII* restriction test.

Because PCR products analysis using a restriction enzyme is thought to allow the possibility of errors in mutant load assessments, secondary to heteroduplex formation during the PCR process [Tanno et al., 1991], we first validated our method of heteroplasmy assessment using mixing experiments and standard curve analysis. Wild-type and mutant plasmids were mixed to generate 10 samples, each of them comprising 10^6^ mtDNA copies, with the m.3243G > A (mutant) target at concentrations ranging from 1 to 100%, which were also used to determine the lowest rate of mutation detection. Each sample was amplified in triplicate, using both “single-cell” and “tissue” methods, and PCR products were submitted to *HaeIII* digestion.

We furthermore ascertained the reliability of our test over a wide range of mtDNA copies, from 10^2^ to 10^6^, encompassing the mtDNA copy number usually present in isolated cells such as lymphocytes, blastomeres, and oocytes.

Single cells and embryos were transferred to 3 µl alkaline buffer [Cui et al., 1989] and lysed by 10-min heating at 65°C. PCR amplification was carried out using the forward primer (5′-TGAGTTCAGACCGGAGTAATC-3′) and the reverse fluorescent primer (^*^) (5′-(6-Fam) CTTAACAACATACCCATGGC-3′). Separate experimental conditions were devised for whole tissue sample and single-cell/early embryo analyses.

Tissue sample DNA (1 ng) was amplified in a 30-µl reaction volume containing 1.5 U Expand Taq DNA Polymerase, 10 × PCR buffer 2 (3 µl; Roche Diagnostics, Mannheim, Germany), 0.5 µM of each primer (Proligo, Paris, France), and 2 mM dNTP mix (Roche Diagnostics, Germany). Initial denaturation was carried out at 97°C for 7 min 20 sec followed by 20 cycles (97°C for 20 sec, 60°C for 30 sec, 68°C for 1 min 15 sec), and final extension of 7 min at 68°C.

For a single-cell or embryo analysis, the PCR reaction contained 3 µl of lysis buffer (lysed cell or negative control), each primer at 0.5 µM, master mix 2 × (12.5 µl, Qiagen Multiplex PCR kit, Qiagen S.A, Courtaboeuf, France), and double-distilled water up to a 25-µl final volume. PCR programs were 15 min of denaturation at 95°C, followed by 25, 26, 27, and 30 PCR cycles for whole embryos, trophoblastic cells, blastomeres, and amniocytes, respectively, consisting of 30 sec at 94°C, 90 sec at 60°C and 60 sec at 72°C, with a 30-min final extension at 60°C.

The resulting 251-bp fluorescent PCR products (1 µl) were digested for 3 hr using 10 units of *HaeIII* and subsequently submitted to electrophoresis using an automated genetic analyser ABI3130 (Applied Biosystems, Norwalk, CT). Digestion generated 73-bp and 170-bp fluorescent fragments for the mutant and wild-type species, respectively. Results were analysed with the Genescan and Genotyper software (Applied Biosystems). The mutant load was calculated by dividing the mutant peak area (73 bp) by the sum of normal (171 bp) and mutant (73 bp) peak areas. Crosshybridization of oligonucleotide primers to nuclear DNA was ruled out by PCR amplification on mtDNA-less *Rho0* cells [Parfait et al., 1998].

#### Ascertainment of the fetal origin of the CV/AF cells

The fetal origin of single cells collected from CV, AF1, and AF2, was ascertained by simultaneous analysis of parental and embryofetal DNA using nuclear (CA)_n_ microsatellite markers (D6S436, D19S559, D19S559, D16S3395, D6S436, and DXS1073 for fetus 2a, 5a, 5b, 6, 8, and 12b, respectively), enabling to check for biparental contribution to the cell genotype, and the absence of fetal cell contamination by maternal DNA [Gigarel et al., 2004].

Amplification of microsatellite markers in single cells required nested PCR. The first “outer” PCR enabled simultaneous amplification of the MELAS mutation and an appropriate microsatellite marker. Briefly, cells were amplified using “single-cell” PCR conditions described above, as a duplex PCR reaction, using 0.5 µM of each primer. 3 µl of the first amplification product were subsequently mixed with 22 µl of “inner” amplification mix, which contained inner fluorescent microsatellite primers at 0.5 µM, master mix 2 × (12.5 µl, Qiagen Multiplex PCR kit), and double-distilled water up to a 25-µl final volume. PCR program was as described above (20 PCR cycles).

### Statistical Analyses

Variance analysis and calculation of inter or intra class correlation coefficients were used to analyze quantitative traits. Qualitative traits were analyzed using a chi-square test. The 95% confidence intervals of the measurements from patient 2 were made by fitting a Kimura distribution [Wonnapinij et al., 2008] to the 35 mutation level measurements from this patient, and then drawing 10,000 simulated sets of 35 measurements from this Kimura distribution to estimate the confidence intervals due to sample size effects [Wonnapinij et al., 2010].

## Results

### Sensitivity, Linearity, and Reproducibility of Mutant Load Quantification

**Figure 1:**
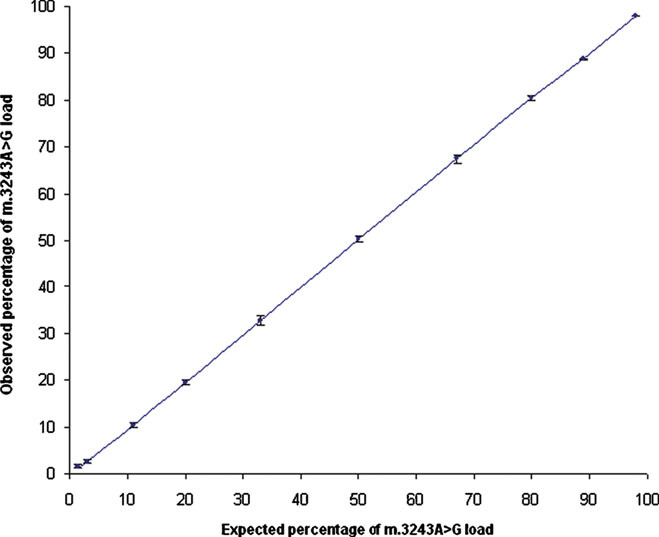
Standardization of m.3243A > G load assessment. The rate of m.3243A > G heteroplasmy was assessed by semiquantitative fluorescent PCR using 10 samples containing various proportions of mutant plasmid. Each sample comprised 10^6^ mtDNA copies. Each value is the mean of three independent experiments. Bars depict standard deviations. WT: wild-type DNA.

Mixing various proportions of wild-type and mutant plasmids enabled us to establish that the measured level of heteroplasmy was a linear function of the expected ratio (Fig. 1). The correlation coefficient for the observed versus expected proportion of mutant was 0.99 for both “single-cell” and “tissue” conditions. Triplicate experiments yielded to similar results (SD<1.7%), and mutant molecules could be detected in a proportion as low as 2% of the total mtDNA amount, thus validating reproducibility and sensitivity of the assay. These results were got with a 10^6^ mtDNA copy number.

To demonstrate an insensitivity to template input amounts for this particular method, we ascertained the reliability of our test over a wide range of mtDNA copies, from 10^2^ to 10^6^. Results are displayed in Supp. Figure S1.

### Analysis in Carrier Women

Mutant loads from the eight carriers applying for MELAS PND/PGD (family 1 to 8; Table 1) ranged from 5 to 40%, 8 to 50%, and 15 to 80%, in leucocytes, oral mucosa cells, and urinary tract cells, respectively. The lowest and highest values were consistently found in white blood cells (WBCs) and urinary tract cells, respectively, as expected from previous reports [Chinnery et al., 1997, 1999; Frederiksen et al., 2006; Whittaker et al., 2009].

**Table 1:** Maternal m.3243A > G Mutant Load

		Mutant load (%)		
		
	Patient	WBC	OMC	UTC
**A**	1	20	30	65
	2	20	27	30
	3	20	40	50
	4	7	8	15
	5	5	10	35
	6	30	50	80
	7	30		70
	8	40		55
**B**	9	39	48	
	10	20		
	11	63		
	12	34	45	80
	13	21		

**A**, Patients analyzed in this study. **B**, Patients reported elsewhere: [9] Matthews et al., 1994, [10] Cardaioli et al., 2000, [11] Chou et al., 2004, [12,13] our group [Bouchet et al., 2006]. WBC, white blood cells; OMC, oral mucosa cells; UTC, urinary tract cells.

Mutant load assessment in single lymphocytes from five carriers showed large intercellular variations, ranging from 0 to 81%, 0 to 81%, 0 to 95%, 0 to 21%, and 0 to 100% in patients 1, 2, 3, 5, and 14, respectively. When pooling values from all cells analyzed for a given individual, average mutant load was very close to that achieved from the overall lymphocyte extract recovered from a 10-ml blood sample.

Comparison of the mean heteroplasmy rate vs one standard deviation of the heteroplasmy distribution at the single-cell level for each of the five lymphocyte pools (16.2 ± 8.6%) showed a 0.92 correlation coefficient (*P*<0.05) with a positive slope (1.14 ± 0.3) (Table 2 and Supp. Fig. S2).

**Table 2:** m.3243A > G Mutant Load in Single Cells of Various Origins

	Lymphocytes	Syncitiotrophoblasts/amniocytes	Muscle fibers^a^
Number of patients	5	5	8
Number of cell pools	5	10	21
Total cell number	84	147	341
Heteroplasmy level (mean ± SD)	16.2 ± 8%	34.3 ± 13%	78.5 ± 17%
Correlation coefficient (p)	0.92 (<0.05)	0.81 (<0.05)	0.84 (<0.01)
Slope ± SD	1.14 ± 0.28	0.13 ± 0.03	−0.38 ± 0.05

a Calculation from data by Tokunaga et al. [1994]; Petruzzella et al. [1994]; Silvestri et al. [2000].

### Analysis in Day 3 to Day 5 Embryos

Thirty-eight embryos from two unrelated heteroplasmic women were analyzed. The m.3243A > G mutant load was assessed in 31 whole embryos and 70 single blastomeres (Table 3). Exogenous DNA contamination of the embryonic sample was never detected in any experiment.

**Table 3:** m.3243A > G Mutant Load in Preimplantation Embryos

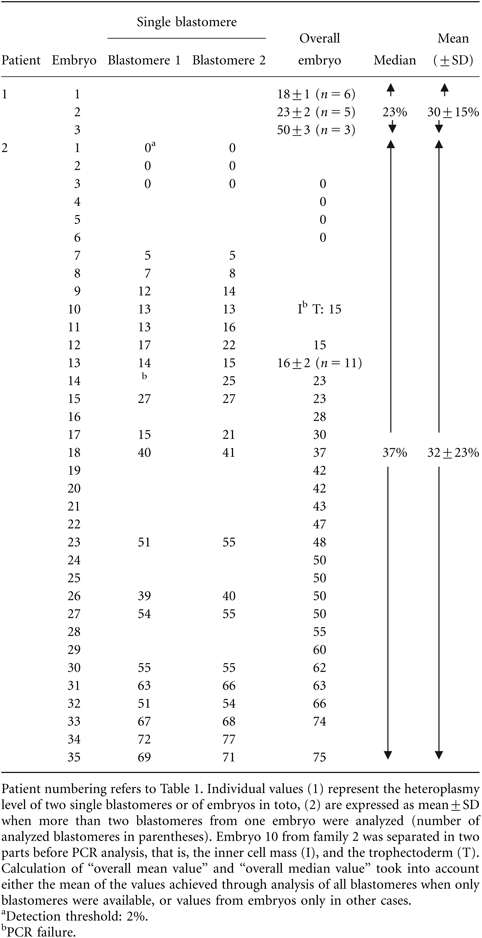

Within the whole cohort of analyzed embryos, six had no detectable mutation (detection threshold: 2%). Taking into account Patient 2 only (35 embryos), the transmission rate was 83%. None of the embryos were homoplasmic mutant. Heteroplasmy levels ranged from 5 to 77%, with an apparently homogeneous dispersion of the mutant rates among embryos. The mutant load value was similar in all blastomeres derived from a single embryo (*n* = 2–11) with a 6% maximal interblastomere variability. The intraclass correlation coefficient (ICC) was equal to 0.994, consistent with the high level of agreement of intraembryo measurements, and contrasting with the interembryo variability. When possible, comparison of heteroplasmy levels between the whole embryo (collected at day 5) and two of his/her blastomeres (collected at day 3) showed a variation rate <7% in 13/16 embryos, and between 10 and 15% in the remaining ones.

When considering all embryos from patient 2, the distribution of the mutation level values was consistent with the distribution predicted from neutral drift theory (*P*-value of 0.53 by KS test) comparing the data against the null hypothesis of the Kimura distribution [Wonnapinij et al., 2008]. When pooling mutant loads measured in each embryo from a given woman, mean values were 30 ± 15% and 32 ± 23% for patients 1 and 2, respectively. Each of these values was close to the mutant load measured in oral mucosa cell DNA from the corresponding woman (30 and 27% for patients 1 and 2, respectively).

Embryo 10 from family 2, harboring a 13% mutant load at day 3, was cultured up to the blastocyst stage (day 5) and separated in 2 parts, namely, the inner cell mass and the trophectoderm. PCR failed to amplify the inner cell mass DNA, while the heteroplasmy level of the trophectoderm was consistent with the level measured in day 3 blastomeres (15 vs. 13%).

### Analysis in Fetuses over 10 GW

Mutant loads were assessed in extraembryonic or embryonic tissues collected between 10 GW to term, in 12 fetuses from 7 carriers (families 2 to 8; Table 4).

**Table 4:** m.3243A > G Load in Extra-Embryonic and Embryo-Fetal Tissues

	Patient	Fetus	CVS	AF1	AF2	Muscle	Liver	Heart	Skin	Kidney	Lung	Gut	Brain	Optic nerve	Thymus	Umbilical cord	Placenta	Cord blood
**A**	2	2a	77 ± 3			75 ± 1.5		74 ± 3.2			74 ± 0						73, 75^a^	
		2b	59 ± 6			42 ± 1		41 ± 2	42 ± 2	43 ± 2	43 ± 0.3						57 ± 2^b^	
		2c		4 ± 0													15 ± 5^c^	5 ± 1
	3	3	17 ± 3	16 ± 0.5													22 ± 6^d^	16 ± 0
	4	4		0														0
	5	5a	20 ± 0.5	15 ± 3	15 ± 2													
		5b		70 ± 1			72 ± 2		73 ± 0	68 ± 2					72 ± 1			
		5c	4 ± 0.6															
	6	6	32 ± 1	28 ± 1														
	7	7a				79 ± 0	78 ± 0.6	77 ± 0.9	78 ± 0.3	79 ± 0	79 ± 0.3					78 ± 1.7	78 ± 1.7	
		7b	79 ± 2															
	8	8	49 ± 0.7															
B	9	9				55 ± 1	52 ± 4	54 ± 3	51 ± 3	56 ± 5		53 ± 4	52 ± 3	51 ± 3			55 ± 3	
	10	10				50	50						50					
	11	11			67													
	12	12a	35	33	29													
		12b	31	23	26													
	13	13a	60	63														
		13b	0	0														
		13c	0	0														

**A**, Fetuses analyzed in this study. Chorionic villi (CVS) were sampled at 10 GW, and amniotic fluids (AF) at 14 (AF1) and 30 (AF2) GW, respectively. The other tissues were sampled at 12 GW (fetuses 2a and 2b), or at 19, 20, 24, and 25 GW for fetuses 5b, 7, 9, and 10, respectively, and at term for fetuses 2c and 3. Cord blood was sampled at birth for fetuses 2c (38 GW), 3 (37 GW), and 4 (39 GW). All assays were carried out in triplicate. Values are expressed as mean ± SD. Placenta values measured on ^a^2, ^b^6, ^c^10, and ^d^18 separate biopsies, respectively (SD: intersample variation). **B**, Fetuses reported elsewhere: [9] Matthews et al., 1994, [10] Cardaioli et al., 2000, [11] Chou et al., 2004, [12,13] our group [Bouchet et al., 2006]. In boldface: patients born after PGD or PND.

One fetus did not carry the mutation in his two tested tissues (amniocytes at 16 GW, and cord blood at birth, fetus 4). As observed in early embryos, no fetus was homoplasmic for the mutant. The mutation load ranged from 4 to 79% in the 11 carriers. There was no substantial variation in mutant loads assessed at various stages of pregnancies until birth (Table 4). When excluding data from extra-embryonic tissues (10 GW trophoblast or at term placenta), m.3243A > G mutant loads were identical in all tested tissues from a given fetus (mean ± SD: 74.6 ± 0.7% [three tissues], 42 ± 0.8% [five tissues], 71 ± 2% [five tissues], and 78 ± 0.9% [seven tissues] for fetuses 2a, 2b, 5b and 7a, respectively). When placenta and other tissues were available at the same term of gestation (three fetuses), values were similar in two fetuses (74.6 vs. 74% and 78 vs. 78% for fetus 2a and 7a, respectively), while fetus' 2b mutant load was higher in placenta than in other tissues (57 ± 2% vs. 42 ± 2%, respectively). Multiple samples in various parts of placenta, aimed at testing the mutant load distribution, did not show any substantial variation in 2, 6, 10, and 18 various loci of fetuses' 2a, 2b, 2c, and 3 placentas, respectively (Table 4), irrespective of the gestation stage (12 GW or at birth).

**Figure 2:**
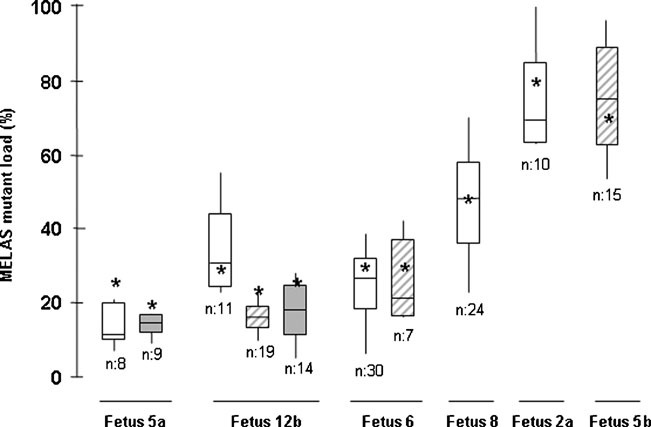
Distribution of m.3243A > G mutation levels across isolated cells. MELAS mutant load was assessed in trophoblastic cells sampled at 10 GW (open boxes) and amniocytes sampled at 14 (hatched boxes) or 30 (filled boxes) GW in six fetuses (numbering refers to Table 4). n: number of analyzed cells. Each box depicts the mean mutant load plus (top bar), and minus 1 standard deviation (bottom bar) for a given cell pool. Whiskers depict maximal (top) and minimal (bottom) values for each sample. Central bar: median. ^*^Mean mutant load assessed on the whole chorionic villi, or amniocyte sample.

Single trophoblastic cells (*n* = 83) and amniocytes (*n* = 64) were isolated from six fetuses to investigate the intercellular mutant load variation (Fig. 2). Simultaneous analyses of parents' and fetal cell DNAs using a polymorphic marker proved the fetal origin and the absence of contamination by maternal DNA of the analyzed cells (not shown). Large intercellular variations were found both in trophoblastic cells (range: 7–21%, 6–38%, 23–55%, 23–70%, and 64–100%, for fetuses 5a, 6, 12b, 8, and 2a, respectively), and amniocytes, either at 14 GW (range: 10–23%, 16–42%, and 52–94%, for fetuses 12b, 6 and 5b, respectively), or at 30 GW (range: 9–17% and 5–28% in fetuses 5a and 12b, respectively). When pooling values from all cells analyzed for a given cell type in a given fetus, the average mutant loads were very close to those achieved from overall chorionic villi sampling (CVS) and amniotic fluid sample (AFS) mutant loads (variation <10%; Fig. 2).

Comparison of the mean heteroplasmy rate versus one standard deviation of the heteroplasmy distribution at the single-cell level for each of the 10 fetal cell pools (34.3 ± 23%) showed a 0.81 correlation coefficient (*P*<0.01) with a positive slope (0.13 ± 0.03) (Table 2 and Supp. Fig. S2).

### Predictive Value of a Prenatal Mutant Load for the Postnatal Outcome

Assessment of the predictive value of a prenatal mutant load for the disease severity in the postnatal period is of crucial importance. A follow-up of our small cohort of patients born after PGD (*n* = 1) or PND (*n* = 9) provided a preliminary indication, even though for ethical reasons we could not check for the presence of the mutation in these asymptomatic children. All these children, harboring mutant loads between 0 and 35% in prenatal period, are indeed healthy at 1 month to 5 years of age (Table 4).

## Discussion

Analysis of tissues and cells from human embryos and fetuses is currently the only approach to get some insight into processes that govern maternofetal transmission of common mtDNA mutations in humans, owing to the numerous technological difficulties met in attempts at creating animal models of mitochondrial disorders [Fan et al., 2008; Inoue et al., 2000]. We used this approach to study the segregation of m.3243A > G mtDNA molecules during human in utero development, thanks to our PND/PGD activities, offered to carriers of this mutation.

These data rely on a robust, specific, and sensitive assay we devised for the quantification of the m.3243A > G MELAS mutation in tissues and single cells.

Although last-cycle labeling PCR indeed is a well-established and agreed-upon standard protocol for the quantification of mtDNA heteroplasmy, a major concern with this approach is the need to add the labeled primer prior to the last PCR cycle. This process indeed runs a significant risk of contamination of both the endogenous PCR product by exogenous DNA, and of the laboratory dedicated to single-cell analyses by the endogenous PCR product. Such a risk, being a minor one when amplifying a large amount of DNA, becomes a major hurdle to the use of this method, when applied to single-cell analysis.

We ascertained the reliability of our test over a wide range of mtDNA copies, from 10^2^ to 10^6^. This range encompassed the mean value of mtDNA copies in lymphocytes (around 150 copies) [Maagaard et al., 2006; Urata et al., 2008', blastomeres (11,000–449,000 copies) [Lin et al., 2004], and oocytes (200,000–800,000 copies) [Craven et al., 2010]. Our results demonstrated an insensitivity to template input amounts for this particular method, thus validating the results of the experiments carried out on various cells and tissues.

### Analysis in Day 3 to Day 5 Embryos

To our knowledge, data on the presence of the m.3243A > G mutation at the day 3 stage of embryonic development have not been reported so far. This mutation was found in 32 of 38 embryos (84%) from two unrelated carriers. This transmission rate is in agreement with a previous report on the presence of m.3243A > G in adult carrier's primary oocytes [Brown et al., 2001], whose 74 of 82 oocytes (90%) were heteroplasmic. A woman carrying the pathogenic mutation may thus have mutation-free embryos, advocating PGD as an appropriate procedure for preventing recurrence of affected offspring in carriers, at least in the mutant load range reported in this study.

Mutant DNA molecules were constantly found to be equally distributed among the various blastomeres of a carrier embryo until day 5, in agreement with data on mtDNA polymorphisms achieved in mouse [Dean et al., 2003] and human embryos [Marchington et al., 2010], as well as on the m.8993T > G (NARP) mutation in human [Steffann et al., 2006]. Single-cell analysis thus reflects the whole embryo mutant load, supporting relevance of the usual PGD procedure for assessment of the overall embryo genotypic status. It is, however, worth noting some occasional variability (up to 15%) in mutant load between single-cell measurements and the whole embryo. This discrepancy could have arisen from either a technical artifact (such as partial loss of mitochondrion during whole embryo recovery) or physiological distortion in m.3243A > G segregation from day 3 (blastomere sampling stage) to day 5 (whole embryo recovery stage). This last hypothesis is unlikely because mutant load values were very close in day 5 trophectoderm and day 3 single blastomeres in the only embryo where both tissues were available (Table 3), additionally indicating that the mtDNA molecule segregation is not skewed across embryonic and extraembryonic cells (trophectoderm) at this developmental stage. Finally, inter blastomere stability of the mutant load suggests that embryonic heteroplasmy determined from a single-cell analysis provides an accurate estimate of the whole embryo mutant load, as previously reported for other mtDNA variants [Craven et al., 2010; Steffann et al., 2006].

The m.3243A > G mutant load was found to be highly heterogeneous among various embryos from a given individual, ranging from 18 to 50% and from 0 to 77% in patients' 1 and 2 embryos, respectively. These values on “early” embryos are in agreement again with those on primary oocytes from a m.3243A > G carrier, the mutant load of which ranged from 1 to 50% [Brown et al., 2001]. Two conclusions can be derived from these observations.

The first conclusion concerns the mode of transmission of m.3243A > G from the mother to her progeny. The distribution of the mutation level values among patient 2's embryos was consistent with the Kimura distribution calculated from the neutral drift theory. Furthermore, an equal number of her embryos were found to harbor a mutation level greater than the mean value (18 embryos) and less than the mean (17 embryos). Finally, the mean level of heteroplasmy amongst all embryos was very close to the heteroplasmy level seen in the mother (oral mucosal cells and blood, patients 1 and 2). These data argue for random genetic drift as the mechanism governing mtDNA segregation during oogenesis. This observation in humans is in full agreement with the results of a study of heteroplasmy distribution in the progeny of mice carrying apparently neutral BALB/c and NZB mtDNA sequence variants [Jenuth et al., 1996]. Assuming that blastomere analysis provides a direct insight on mature oocyte mutant load, our data, gathered with those of Brown et al., suggest that mtDNA segregation is governed by a random genetic drift mechanism over the whole oogenesis period.

The second conclusion regards the size of the bottleneck operating for m.3243A > G during oogenesis (Table 5). The embryos from patient 2 had a larger normalized variance [Wonnapinij et al., 2010], and therefore a smaller bottleneck parameter, than the oocytes from the individual in Brown's study [Brown et al., 2001]. The confidence intervals did not quite overlap, indicating that there is a real difference between the bottleneck sizes in these two individuals, although this difference is not large. These data therefore suggest that some individual-dependent parameter modulates the bottleneck size for a given mtDNA mutation.

**Table 5:** Comparative Analysis of Mutation Levels in Preimplantation Embryos from Patient 2 and Primary Oocytes from a Published Report

	Patient 2	Brown et al., 2001
Sample size (*n*)	35	82
Mean mutation level (*p*_0_)	33.7% (25.8–42.1%)	12.6% (10–15%)
Mutation level variance (*V*)	0.06 (0.037–0.086)	0.014 (0.009–0.020)
Normalized variance (*V*/(*p*_0_(1 − *p*_0_)))	0.27 (0.18–0.38)	0.13 (0.09–0.17)
Bottleneck parameter (*b*)	0.72 (0.62–0.82)	0.87 (0.83–0.91)
Probability of fixing on wild-type	8.4% (1.5–19%)	14% (6–24%)
Probability of fixing on the mutant	0.4% (0–2.0%)	2 × 10^−7^ (0–1 × 10^−6^)
Probability of having > 60% mutant	17% (7–27%)	0.3% (0.02–0.8%)

Values are given as mean (95% confidence intervals). The distribution of the mutation level values from patient 2 was consistent with the Kimura distribution (*P*-value of 0.53 by KS test).

### Analysis in 10–37 GW Fetuses

Very few data are available on m.3243A > G segregation during fetal development so far. We looked for this mutation in 12 fetuses from 8 carriers, and aggregated these data with those that we (patients 12 and 13; Table 4) [Bouchet et al., 2006] and other groups (patients 9 to 11; Table 4) [Cardaioli et al., 2000; Chou et al., 2004; Matthews et al., 1994] had previously published. Maternal m.3243A > G was passed on to 17 of 20 fetuses (85% transmission rate). Taking embryonic and fetal data together, carriers passed on the mutation to 49 of their 58 offspring (84%; Tables 3 and 4). This transmission risk fits with the MELAS transmission rate calculated from the mutant load determined in buccal mucosa samples from carriers' offspring in the postnatal period [Uusimaa et al., 2007]. It cannot be ascertained whether the three fetuses shown to be mutation-free at 10 GW in the current study were mutation-free embryos initially or alternatively resulted from subsequent loss of mutant mtDNA molecules they carried at early embryonic stage. All fetuses but one (fetus 2c) were indeed conceived out of a PGD procedure, and mutant load values at the day 3 stage were therefore not available. Our data however argue for mutant load stability throughout the entire period of embryofetal development. In fetus 2c, loads in 14-GW amniocytes and in term cord blood were indeed 4 and 5%, respectively, in agreement with those assessed at early embryonic stage (embryos 7 and 11 from patient 2, carrying 5 and 14%, respectively, only one of whom, fetus 2c, developed). We similarly did not find any temporal variation of the MELAS mutant load in multiple samples taken within 10 to 39 GW (Tables 4A and B).

Study of various tissues from six 12–25 GW fetuses (2a, 2b, 5b, 7a, 9, 10) showed a tight intertissue stability in each of them. Mutant load assessed in extra-embryonic (placenta) and embryonic tissues collected at the same gestation term from four fetuses, was found to be similar in three of them (2a, 7a, and 9), being higher in trophoblast (57 vs. 42%) from the remaining one (fetus 2b). Discrepancy between trophoblast and nontrophoblastic fetal tissues did not seem to result from an ascertainment bias secondary to locoregional mutant load heterogeneity within placenta, recently shown to exist [Marchington et al., 2006]. The heteroplasmy level was indeed highly similar in six separate regions of fetus 2b placenta (Table 4). Thus, this placento-fetal discrepancy might be accounted for by selection of a few cells that will form the first source for the placental membranes in the partitioning blastocyst. This observation additionally emphasizes that analysis of single CVS sample, carried out in a PND frame, may fail to assess the fetal mutant load reliably, as recently reported [Marchington et al., 2010].

The observation of tight mutant load stability, both across tissues and with time, in all tested fetuses, suggests that mtDNA segregation is mainly governed by random genetic drift in somatic tissues throughout the entire period of human embryofetal development. Furthermore, as already noted in embryos, none of the 20 fetuses analyzed so far (Table 4) was found to carry a heteroplasmy rate exceeding 80%, over a wide range of maternal mutant loads (from 5% in WBCs to 80% in urinary tract cells). Whether such a 80% value has a biological significance (i.e., a hypothetical selection against highly mutated embryos/fetuses) remains speculative. Because mutations such as m.3243A > G exhibit a relatively normal pattern of distribution around the maternal mean, it would be unlikely, given the relatively small sample size and the mean maternal mutant load value in majority below 40% (in blood) in our series (Table 1) to find embryos with greater than 80% heteroplasmy, even in the absence of selection.

We attempted to establish at the single-cell level the basis of the mutant load stability observed across fetal tissues. We thus analyzed 147 cells distributed into 10 pools of trophoblastic or amniotic cells (7–30 cells per pool) collected in six unrelated fetuses within the 10–30-GW period (Fig. 2). Except for one homoplasmic mutant cell (fetus 2a), all cells were heteroplasmic with a mutant load ranging from 5 to 95%. This observation is in line with the few studies available on MELAS single cells collected during the postnatal life, that indeed failed to detect mutant loads over 92% and 98% in lymphocytes [Saitoh et al., 1999] and muscle fibers [Silvestri et al., 2000], respectively. These data argue that, over a critical threshold of heteroplasmy, the resulting impact on respiratory chain function promotes fetal cell death [Sasarman et al., 2008].

When pooling values from all cells belonging to a given pool, mutant load dispersion ranged from 10 to 47% (Fig. 2). The frequency distribution of a single-cell mutant load within each of the 10 cell pools corresponded to a binomial distribution, with a median value identical or very close to the mean value of heteroplasmy, in keeping with the random genetic drift mechanism. We subsequently looked for an optional relationship between a mutation rate at the tissue (chorionic villi or amniocytes) level and the amplitude of mutant load dispersion at the single-cell level in this tissue (Supp. Fig. S2). A previous study using adult carrier lymphocytes had indeed suggested that the higher the proportion of mutated mtDNA molecules, the wider the dispersion of heteroplasmy level [Saitoh et al., 1999]. Although the number of single cells analyzed was low in each pool, we however assumed that each of the 10 cell pools was fairly representative of the whole tissue sample, based on mean mutant load variation consistently less than 10% between the cell pool and the whole sample. It clearly appeared that the higher the tissue mutant load, the larger the heteroplasmy dispersion in trophoblastic and amniotic cells, even if the low number of analyzed cells precluded any firm conclusion on the biological relevance of this observation. We then attempted to evaluate whether such a hypothetical relationship can be considered in various tissues from m.3243A > G carriers, collected in prenatal and postnatal periods as well. We considered that Saitoh's study was exposed to some bias in heteroplasmy assessment, due to the complex experimental procedure of this study (first PCR, electrophoresis gel extraction of the PCR products, second PCR, PCR products enzymatic digestion, Southern blotting, and bioimager analysis). Using DNA from five adult carrier females, we therefore recapitulated Saitoh's study with our own method of mutant load assessment, in an effort to homogenize our data. We thus found a statistically significant correlation between the mean mutant load in each of the five lymphocyte pools and the heteroplasmy distribution at the single-cell level. Interestingly, comparison of our data in lymphocytes and fetal cells showed a marked difference between curve slopes for a same range of heteroplasmy, arguing for some tissue-dependence of mtDNA segregation at the cell level (Supp. Fig. S2). These data contrast with the few available data of the literature, regarding single muscle fibers in adult carriers. The latter indeed showed that, the higher the mean mutant load of a cell pool, the lower the heteroplasmy distribution at the cell level [Petruzzella et al., 1994; Silvestri et al., 2000; Tokunaga et al., 1994]. However, the range of mutant loads does not overlap between skeletal muscle (60–100%) and the other tissues (5–60%). Thus, assuming that the apparent relationship between mean mutant loads at a whole sample level and mutant load dispersion at the single-cell level makes biological sense in all these tissues, it would remain to be seen whether the proposed tissue dependance of such correlations is valid throughout the 0–100% range of heteroplasmy.

### Comparison of Prenatal and Postnatal Data

It is difficult to reconcile the prenatal intertissue stability observed in this study, and the tissue-dependance of m.3243A > G mutant loads in adult carriers, who harbor heteroplasmy rates almost constantly higher in skeletal muscle, urinary epithelial tract cells, and hair follicles, than in white blood cells [Chinnery et al., 1999; Frederiksen et al., 2006; Whittaker et al., 2009].

This tissue dependance has been suggested to result from a replicative disadvantage of cells harboring a high mutant load, thus promoting “mutation epuration” from fast regenerating tissues. Should such a biological process operate prenatally, it could be missed out within the “short” course of a pregnancy, by reference to the very slow decrease of mean mutation level in adult white blood cells [Rajasimha et al., 2008], thus reflecting the apparent intertissue mutant load stability throughout the prenatal period. Alternatively, cell proliferation rate would be identical among all tissues throughout intrauterine life, and would become tissue dependent from a fairly late postnatal period. A similar value of mutant load (65–70%) has indeed been reported in amniocytes at 21 GW, and in peripheral blood and hair follicles sampled in a 4-year-old carrier [Chou et al., 2004].

### Comparison of mtDNA Metabolism in m.3243A > G (MELAS) and m.8993T > G (NARP) Mutations Throughout Human Embryofetal Development

**Figure 3:**
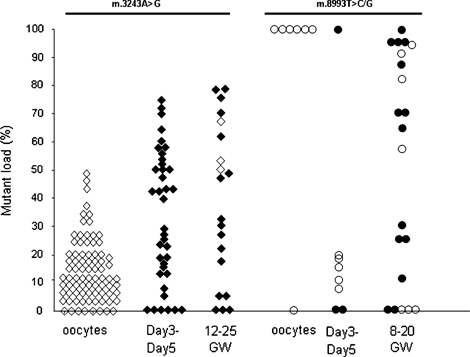
Comparative distribution of m.3243A > G and m.8993T > G/C mutant loads in mature oocytes, blastomeres, and fetal tissues. ⋄ = m.3243A > G (MELAS syndrome); ○ = m.8993T > G/C (NARP syndrome). Scatter plots are drawn from personal (black symbols) and literature data (white symbols) [Blok et al., 1997; Brown et al., 2001; Cardaioli et al., 2000; Chou et al., 2004; Ferlin et al., 1997; Harding et al., 1992; Leshinsky-Silver et al., 2003; Matthews et al., 1994; Pettman et al., 2007; Tajima et al., 2007; White et al., 1999]. Each point depicts one oocyte or one individual (embryo or fetus). All oocyte values were from a single carrier of m.3243A > G [Brown et al., 2001] or m.8993T > G [Blok et al., 1997]. When several values were available for one individual, the average heteroplasmy value was selected. Both day 3 and 14 GW values were available (and therefore depicted) for one “NARP” (0% and 0%) and one “MELAS” individual (5% and 5%) only. All other day 3 to day 5 and 8–25 GW (gestation weeks) values were from distinct individuals.

Comparing mtDNA segregation data throughout human embryofetal development for two different mtDNA mutations, namely, m.3243A > G/MELAS and m.8993T > G/NARP, highlights striking differences (Fig. 3).

Although all MELAS embryos herein reported, originating from 20%-mutant load carriers, are mutation-free or heteroplasmic up to 75% with an apparently random distribution of heteroplasmy, the only two reports on m.8993T > G at day 3 to day 5 embryonic stage indicated that the three NARP embryos from a 18% mutant load carrier were either wild-type or mutant homoplasmic [Steffann et al., 2006]; the five remaining ones carrying low levels of heteroplasmy (4–22%) [Tajima et al., 2007]. Although the low number of embryos analyzed so far precludes drawing firm conclusion on a putative difference of segregation between m.8993T > G and m.3243A > G molecules during oogenesis, these apparently different patterns of segregation are, however, substantiated by the few available analyses of primary oocytes from carriers of these mutations [Blok et al., 1997; Brown et al., 2001]. A wild-type (1/7) or mutant homoplasmic state (6/7) was indeed the rule in the seven NARP primary oocytes from a 50% mutant load carrier, contrasting with the presence of heteroplasmy in 90% of 82 primary oocytes in a m.3243A > G carrier with a 8% mutant load in WBC. It can be speculated from these data that bottleneck would be of different size between m.3243A > G and m.8993T > G.

Whether a purifying selection operates in the human germline against m.8993T > G and m.3243A > G is a matter of debate. Our data in early NARP embryos [Steffann et al., 2006], those from the analysis of primary oocytes in a m8993T > G carrier [Blok et al., 1997], as well as our previous report on two fetuses with a mutant load over 85% from two m8993T > G carriers with a 30% mutant load in WBCs [Steffann et al., 2007], clearly argue against such a negative selection for this mutation in human. As mentioned above, no conclusion can be drawn at this point on the occurrence of a purifying selection against m.3243A > G in the human germ line, owing to the limited number of data we achieved, all drawn from low (20%) mutant load carriers. It has, however, to be emphasized that we never observed any fetus with a mutant load over 80% among the eight pregnancies from six m.3243A > G carriers with a WBC mutant load equal or even higher than 30% (patients 6–9, 11, 12; Table 1). These data are difficult to reconcile with those from murine germline segregation studies, indicating that mutations in protein coding genes of mtDNA are strongly selected against, whereas mutations affecting tRNA genes (such as m.3243A > G) are largely refractory from such a process [Fan et al., 2008, Stewart et al., 2008].

Regarding the somatic tissue development, we previously showed the existence of fetuses carrying m.8993T > G with various degrees of mutant loads, from 0 to 100%, over the 10-GW stage of pregnancy until delivery [Steffann et al., 2007]. m.8993T > G and m.3243A > G mutant loads remain at a steady-state level with time or across various tissues, thus supporting a random segregation of mutant mtDNA molecules throughout somatic embryo-fetogenesis irrespective of the mutation type.

In conclusion, one can speculate that the bottleneck size, and possibly, the mutant load threshold critical for embryo/fetus survival, varies among mtDNA mutations, thus hampering genetic counselling and PND/PGD procedures in mitochondriopathies resulting from “private” mtDNA mutations. To test this speculation, we will need to collect data on mtDNA mutant loads for other rarer mtDNA mutations.
