# Supplementary material for: Segregation of mtDNA Throughout Human Embryofetal Development: m.3243A > G as a Model System
Source: Hum Mutat. 2010 Nov 30;32(1):116–25. doi: 10.1002/humu.21417 (PMC3058134; doi:10.1002/humu.21417)
Supplement: Supplementary file 1 [file humu0032-0116-SD1.pdf]

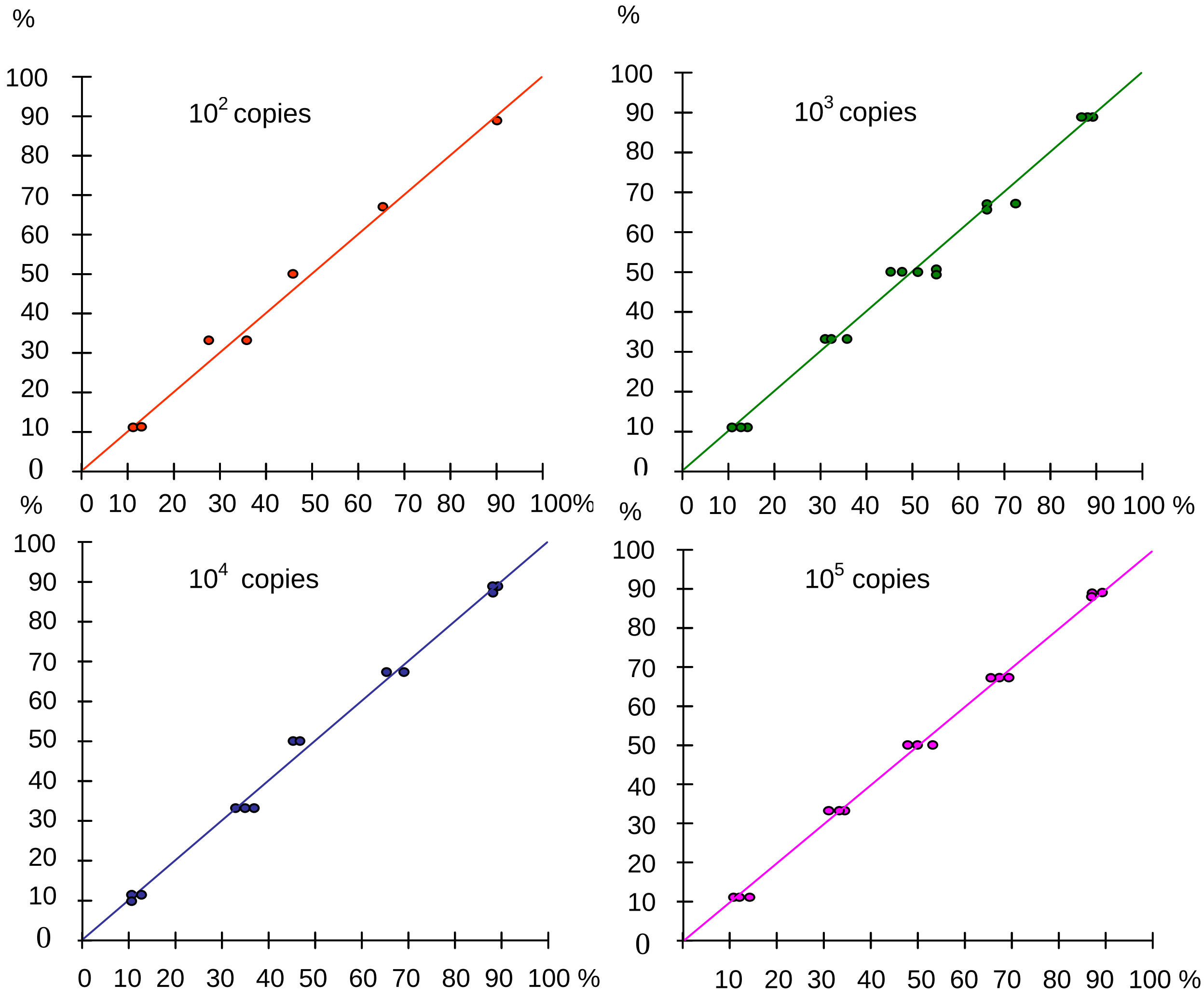

Supp. Figure S1. Standardization of m.3243A>G load assessment over a 10^2-10^5 mtDNA copy range. The rate of m.3243A>G heteroplasmy was assessed by semi-quantitative fluorescent PCR using 5 samples containing various proportions of mutant plasmid (11%, 33%, 50%, 67%, 89%) and 5 different template amounts (10^2, 10^3, 10^4, $10^{5,}$ 10^6). Values achieved with 10^6 copies are displayed as Figure 1. Each dot depicts the result of one experiment. Y axis: expected percentage of m.3243A>G mutant load. X axis: observed percentage of m.3243A>G mutant load.

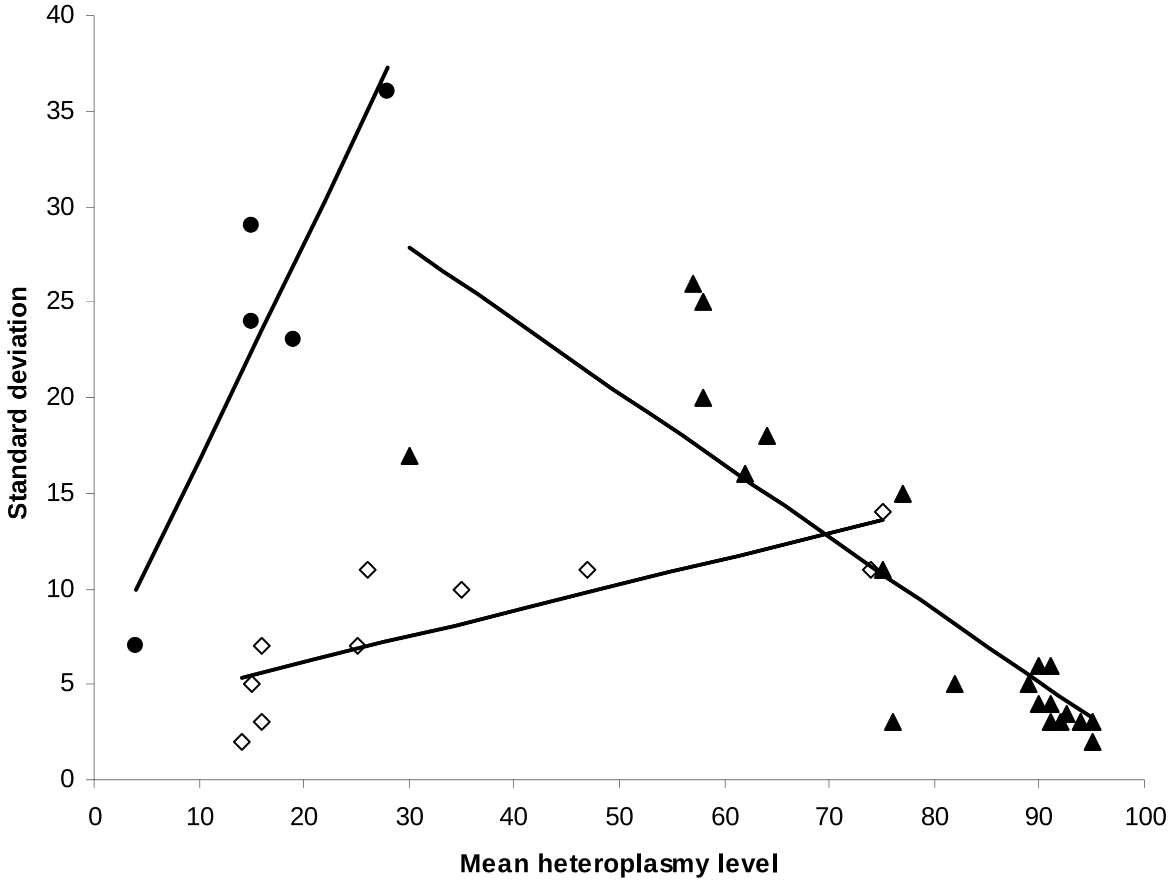

Supp. Figure S2. Distribution of inter cell dispersion of m.3243A>G mutant load according to the heteroplasmy rate of overall cell pools. Data are represented as standard deviation of the mean mutant load vs mean heteroplasmy rate for each pool. ◇ prenatal cell pools: syncitiotrophoblasts and amniocytes (data from Table 2 and Figure 2); Black symbols: postnatal cell pools: ● lymphocytes (data from Table 2), ▲ muscle fibers (data from Petruzella et al., 1994; Tokunaga et al., 1994; Silvestri et al., 2000). Each symbol reflects the analysis of one cell pool for one individual.
